# Effects of energy supplements on the differentiation of skeletal muscle satellite cells

**DOI:** 10.1002/fsn3.2001

**Published:** 2020-12-13

**Authors:** Rina Su, Bohui Wang, Min Zhang, Yulong Luo, Debao Wang, Lihua Zhao, Lin Su, Yan Duan, Luigi Faucitano, Ye Jin

**Affiliations:** ^1^ College of Food Science and Engineering Inner Mongolia Agriculture University Hohhot China; ^2^ Inner Mongolia Vocational college of Chemical Engineering Hohhot China; ^3^ Agriculture and Agri‐Food Canada Sherbrooke Research and Development Centre Sherbrooke QC Canada

**Keywords:** AMP‐activated protein kinase, differentiation, energy supplement, glucose, skeletal muscle satellite cells

## Abstract

To investigate the effects of the activator of AMPK and high glucose on the differentiation of mouse SMSCs, primary SMSCs were isolated from mouse extensor digitorum longus muscle and grown to near confluence (80%). Postconfluent cells were cultured in a growth medium with different inductors: AICAR, glucose, and AICAR mixed with glucose. The specific protein expressions of SMSCs, myoblasts, adipocytes, and brown adipocytes were analyzed on days 0, 3, 5, 7, and 10. The results showed treatment with AICAR in SMSCs markedly activated AMPK phosphorylation (*p* < .05) and increased protein expression of Pax7 and MyoD (*p* < .05), high concentrations of intracellular glucose upregulated UCP‐1 protein expression and enhanced lipid accumulation (*p* < .05), the cowork of AICAR and glucose affected a decrease on MyoD, PPARg, and UCP‐1 expression (*p* < .05) and an increase on Pax7. The present study indicated that the certain energy supplements influence the direction of SMSC differentiation which may contribution on the structure of muscle and meat quality, sequentially.

## Graphical abstract

To investigate the effects of the activator of AMPK and high glucose on the differentiation of mouse SMSCs, primary SMSCs were isolated from mouse extensor digitorum longus muscle and grown to near confluence (80%). Postconfluent cells were cultured in a growth medium with different inductors: AICAR, glucose, and AICAR mixed with glucose. The present study indicated that the certain energy supplements influence the direction of SMSC differentiation which may contribution on the structure of muscle and meat quality, sequentially.

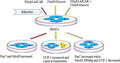

## 1 INTRODUCTION

It has been established that myoblasts and adipocytes arise from multipotent mesenchymal stem cells during the embryonic and fetal stage of mammals (Du & Carlin, 2012). After birth, skeletal muscle satellite cells (SMSCs) which form adult stem cells are located underneath the basal lamina of muscle fibers. These cells have myogenic, adipogenic, and osteogenic differentiation potentials (Tao et al., 2010). Moreover, changes in the proliferation and myogenic differentiation of SMSCs may alter an animal's muscle mass postnatally; also, the number of intramuscular adipocytes appearing as marbled fat can be increased by adipogenic differentiation of SMSCs (Bi & Kuang, 2012; Du & Carlin, 2012). Our former study demonstrated that physical exercise activates SMSCs and myoblast proliferation in longissimus dorsi (LB) of Sunit sheep, resulting in decreased pH and an increase in the yellow color of meat (Su et al., 2019). Therefore, the proliferation of SMSCs may be affected by a variation in extracellular energy.

Adenosine monophosphate–activated protein kinase (AMPK) is a well‐characterized sensor of cellular energy that regulates protein metabolism in skeletal muscle (Lee et al., 2015). AMPK is involved in the regulation of SMSCs during regeneration, which activates the myogenic program to repair damaged myofibers (Shan et al., 2014). For example, it was observed that knocking out the α1 isoform of AMPK resulted in a high, self‐renewal rate, and Warburg‐like glycolysis in SMSCs (Fu et al., 2015). A variety of adenosine triphosphate (ATP)‐consuming stimuli can activate AMPK, such as AICAR, exercise, electrical stimulation, and glucose deprivation (Gordon et al., 2008; Hutber et al., 1997; Salt et al., 1998; Winder & Hardie, 1996). Recently, it is suggested that the AMPK phosphorylation level, as a indicator of energy variation, may altered by exercise, and AICAR as an AMPK activator is involved in the differentiation of SMSCs in vitro. Therefore, it is the purpose of this study to investigate the effects of energy alteration on SMSC differentiation.

Alternatively, a high concentration of glucose not only increases extracellular energy but also has been confirmed to induce adipogenic differentiation in mesenchymal stem cells (Aguiari et al., 2008; Ronningen et al., 2015; Tao et al., 2010). Aguiari et al. (2008) found that primary cells derived from adipose tissue or skeletal muscle can differentiate into adipocytes when cultured in high glucose to form viable and vascularized adipose tissue when implanted in vivo. Therefore, it is possible to induce the direct conversion of myoblasts to adipocytes by increasing the concentration of plasma glucose. This would increase intramuscular adipogenic differentiation of SMSCs resulting in marbling (Guillet‐Deniau et al., 2004). It has been demonstrated that both AMPK activity and plasma glucose concentration contribute to muscle mass in humans and rodents (Guillet‐Deniau et al., 2004; Theret et al., 2017). 5‐Amino‐1‐β‐d‐ribofuranosyl‐imidazole‐4‐carboxamide (AICAR) is one of the mainly activators which can be used as an experimental tool to activate AMPK (Merrill et al., 1997). In this respect, chronic treatment with AICAR to activate AMPK in muscle was reported to eventually result in glycogen accumulation (Winder & Holmes, 2000). Furthermore, glucose suppresses AMPK activation in isolated rat skeletal muscle and attenuation of exercise‐induced AMPK‐α2 activation in human muscle by oral ingestion has been reported (Akerstrom et al., 2006). Thus, the objective of this study was to assess the singular effects of AMPK activity and high glucose induction and their combination on the differentiation of SMSCs. In this respect, this study intended to explore the influence of energy supplementation on the multipotency differentiation of SMSCs, which may contribute to the development of postnatal muscle mass and intramuscular fat and thus improve meat quality.

## 2 MATERIALS AND METHODS

### 2.1 Isolation of primary mouse muscle satellite cells

Satellite cells were isolated from the hind limb muscles of three 2‐week‐old mice as previously described (Pasut et al., 2013) with some modifications. The muscles were dissected and minced to release cells by digestion in buffer containing a collagenase/dispase solution. Fast‐attaching nonmyogenic cells were depleted by repeated plating. Primary satellite cells displayed as fusiform shape and were plated in growth medium (GM) that consisted of DMEM/F‐12 (Gibco, Thermo Fisher) with 20% fetal bovine serum (Sijiqing), 10% horse serum (Solarbio), and 1% antibiotic mixture (Gibco, Thermo Fisher). The cells were incubated in a 37℃ standard cell culture incubator with 5% CO_2_ (Thermo Fisher).

### 2.2 Differentiation assay

The SMSCs were grown in GM, which was changed every day until confluence to 80%. The postconfluent cells were cultured in four differentiation media: GM as control group (CG); GM + 300 µM AICAR (Ark Pharm, Montluçon, France) as the AICAR group (AG); GM + 25 mM glucose as the glucose group (HG); and GM + 300 µM AICAR + 25 mM glucose as the mixture group (AG + HG). Each group was prepared in triplicate, and the medium was changed every 48 hr until day 10. The cell samples were harvested on 0 day, 3 days, 5 days, 7 days, and 10 days for immunohistochemistry, Western blot, and Oil Red O analyses.

### 2.3 Immunohistochemistry analysis

The cells on day 0 (undifferentiated) and day 10 (differentiated) were trypsinized and grown in six‐well plates for immunohistochemistry analysis. Briefly, after the culture medium was completely removed, the cells were fixed in 4% paraformaldehyde for 10 min, permeabilized with 0.5% Triton X‐100 for 5 min, blocked with BSA (Bovine Serum Albumin) at 5 mg/ml, and incubated with the primary antibody Pax7 (monoclonal goat anti‐mouse, 1:1,000; Abcam) overnight at 4°C. The cells were stained with corresponding secondary antibodies (mouse secondary antibody, 1:100; ProteinTech Group, Wuhan, China) for 1 hr at room temperature. The nuclei were counterstained with 10 µl DAPI (Thermo Fisher) for 5 min at room temperature. Images were taken with a fluorescence microscope (Olympus, Tokyo, Japan) and presented with Image‐Pro Plus software (Media Cybernetics, Inc.).

### 2.4 Protein extraction and western blot analysis

Postconfluent cell samples on days 0, 3, 5, 7, and 10 were dislodged from the plates by lysis using a 100:1 mixture of RIPA (radio immunoprecipitation assay) buffer and PMSF (phenylmethylsulfonyl fluoride) protease inhibitor (Beyotime, Shanghai, China). After homogenization, the samples were centrifuged (17950 *g* for 10 min at 4°C). The concentration of total protein in the supernatant was detected using a BCA (bicinchoninic acid) kit (Beyotime institute of biotechnology). A sample mixture of 5‐fold concentrated buffer sample and homogenized proteins was boiled for 5 min; then, the sample mixture was separated by SDS‐PAGE (sodium dodecyl sulfate–polyacrylamide gel electrophoresis) using a 10% polyacrylamide separating gel and a 5% stacking gel. The proteins were transferred to a PVDF (polyvinylidene fluoride) membrane using a Mini Trans‐Blot Cell (Bio‐Rad) and incubated overnight at 4°C with the primary antibody (Pax7; MyoD: polyclonal goat anti‐rabbit, 1:500; Santa Cruz Biotechnology, Dallas, TX, USA; PPARg: polyclonal goat anti‐rabbit, 1:1,000; ProteinTech Group, Rosemont, IL, USA; UCP‐1 [uncoupled protein‐1]: polyclonal goat anti‐rabbit, 1:1,000, Abcam; AMPK: polyclonal goat anti‐rabbit, 1:1,000, Cell Signaling Technology, Danvers, MA, USA; p‐AMPK at Thr172: monoclonal goat anti‐rabbit, 1:1,000, Cell Signaling Technology; and β‐actin: monoclonal goat anti‐mouse, 1:3,000, Santa Cruz Biotechnology) and for 1 hr at room temperature with the secondary antibody (mouse anti‐goat, 1:1,000; ProteinTech Group, Wuhan, China; rabbit anti‐goat, 1:1,000; ProteinTech Group). The blots were imaged with an E‐Gel imaging system (Thermo Fisher, Beijing, China), and band intensities were analyzed using ImageJ software (https://imagej.nih.gov/ij/). Band density was normalized by β‐actin content.

### 2.5 Oil Red O staining of intracellular lipids

The accumulation of triglycerides on days 0, 3, 5, 7, and 10 in three groups was visualized by staining the cells with Oil Red O (Sigma). The cells were transplanted to a 24‐well plate, cultured in 37°C under 5% CO2 incubator for 2 hr, and then fixed with 10% formalin solution (Sinopharm Chemical Reagent Co.) for 20 min. The cells were stained with Oil Red O working solution for 10 min, rinsed with 60% isopropyl alcohol (Sinopharm Chemical Reagent Co.), and microphotographed. Oil Red O concentration was measured spectrophotometrically at 510 nm using a microplate reader (Synergy H1; BioTek, Thermo Fisher).

### 2.6 Statistical analysis

Treatment time intervals were analyzed by SPSS software (IBM Corp.). The normality of data distribution and the homogeneity of variance were tested with the Shapiro–Wilk test and Levene's test, respectively. If the ANOVA assumptions were violated, a nonparametric test or a Welch correction was applied when appropriate. The results were expressed as means ± SE. Differences among means with *p* < .05 represented statistically significant differences.

## 3 RESULTS AND DISCUSSIONS

### 3.1 Effects of AMPK activator on differentiation of skeletal muscle satellite cells

**FIGURE 1:**
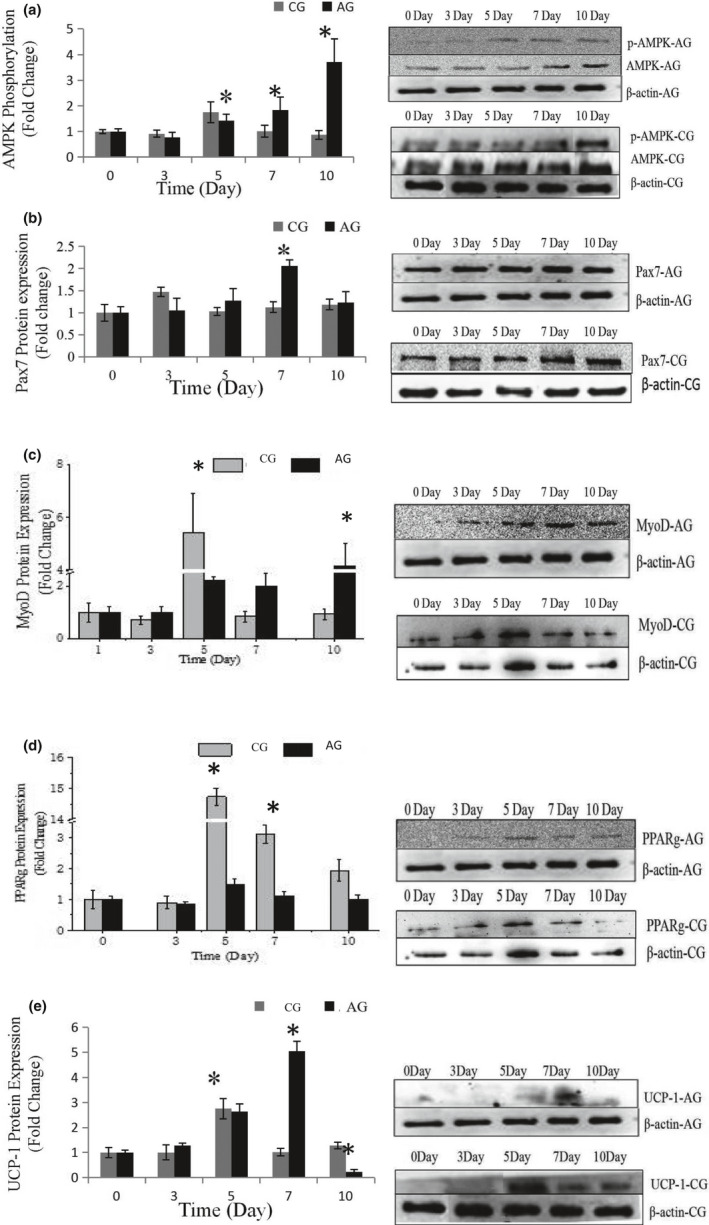
Effects of 300 μM AICAR on the relative protein expression during the process of differentiation of SMSCs. Data are presented as mean ± SE. *Indicates a significant difference (*p* < .05) within an item compares with the undifferentiated cells (0 day) in A300 and Control, respectively. A300: growth medium with 300 μM AICAR group; Control: growth medium as control group

**FIGURE 2:**
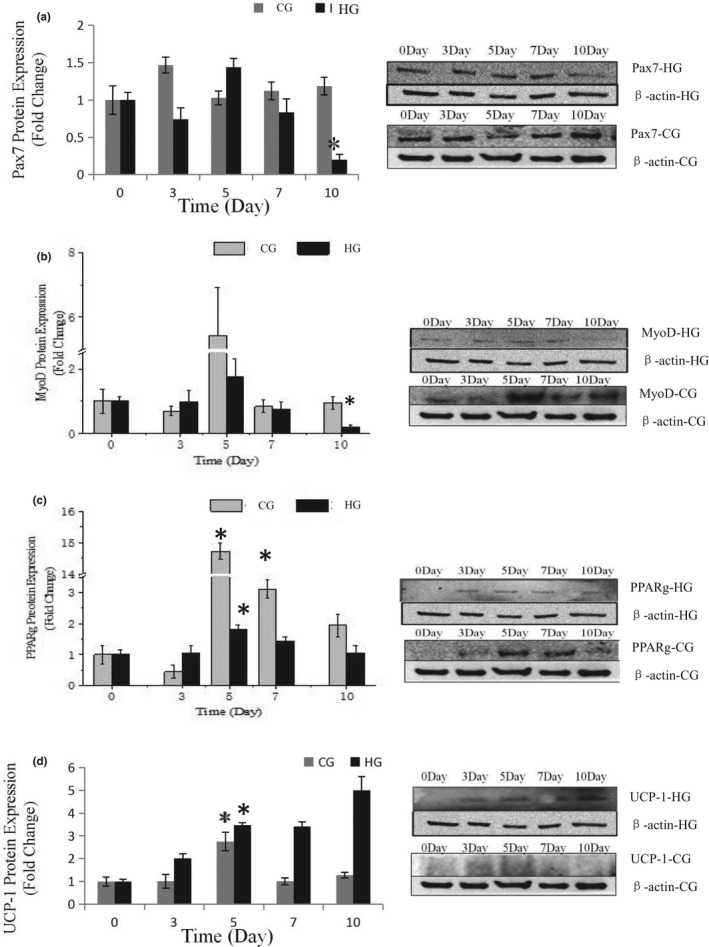
Effects of 25 mM glucose on the relative protein expression during the process of differentiation of SMSCs. Data are presented as mean ± SE. *Indicates a significant difference (*p* < .05) within an item compares with the undifferentiated cells (0 day) in H25 and Control, respectively. H25: growth medium with 25 mM glucose group; Control: growth medium as control group

**FIGURE 3:**
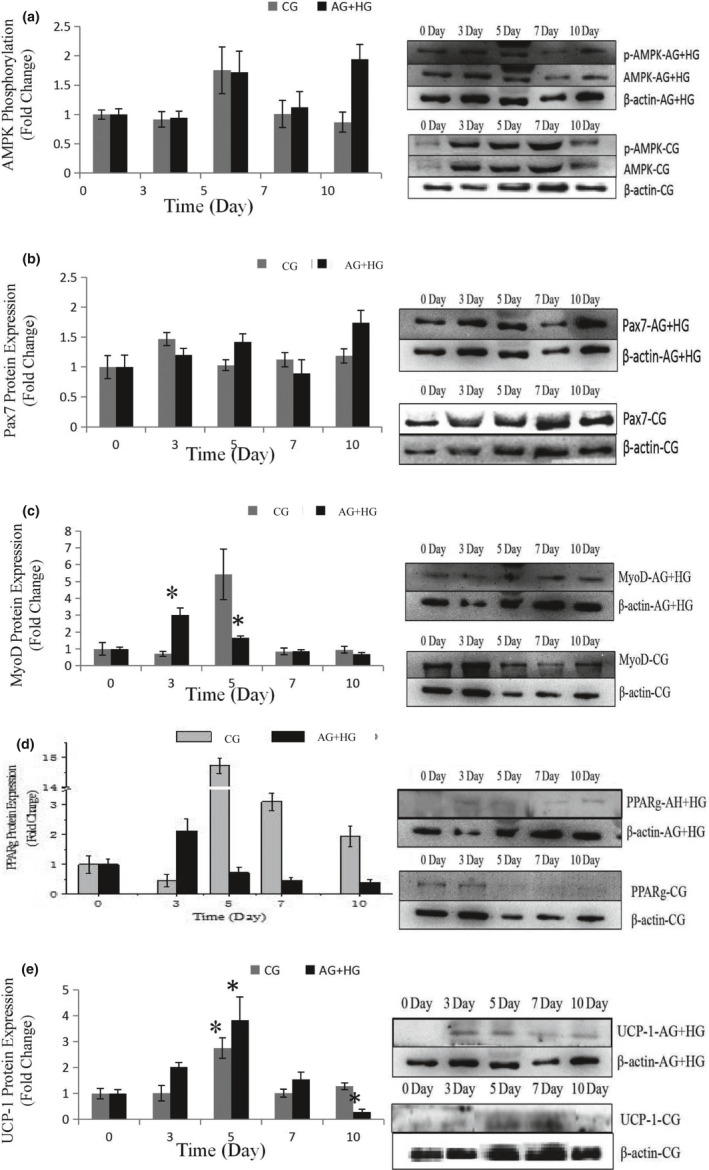
Effects of the cowork of 300 μM AICAR and 25 mM glucose on the relative protein expression during the process of differentiation of SMSCs. Data are presented as mean ± SE. *Indicates a significant difference (*p* < .05) within an item compares with the undifferentiated cells (0 day) in H25‐A300 and Control, respectively. H25‐A300: growth medium with 300 μM AICAR and 25 mM glucose group; Control: growth medium as control group

**FIGURE 4:**
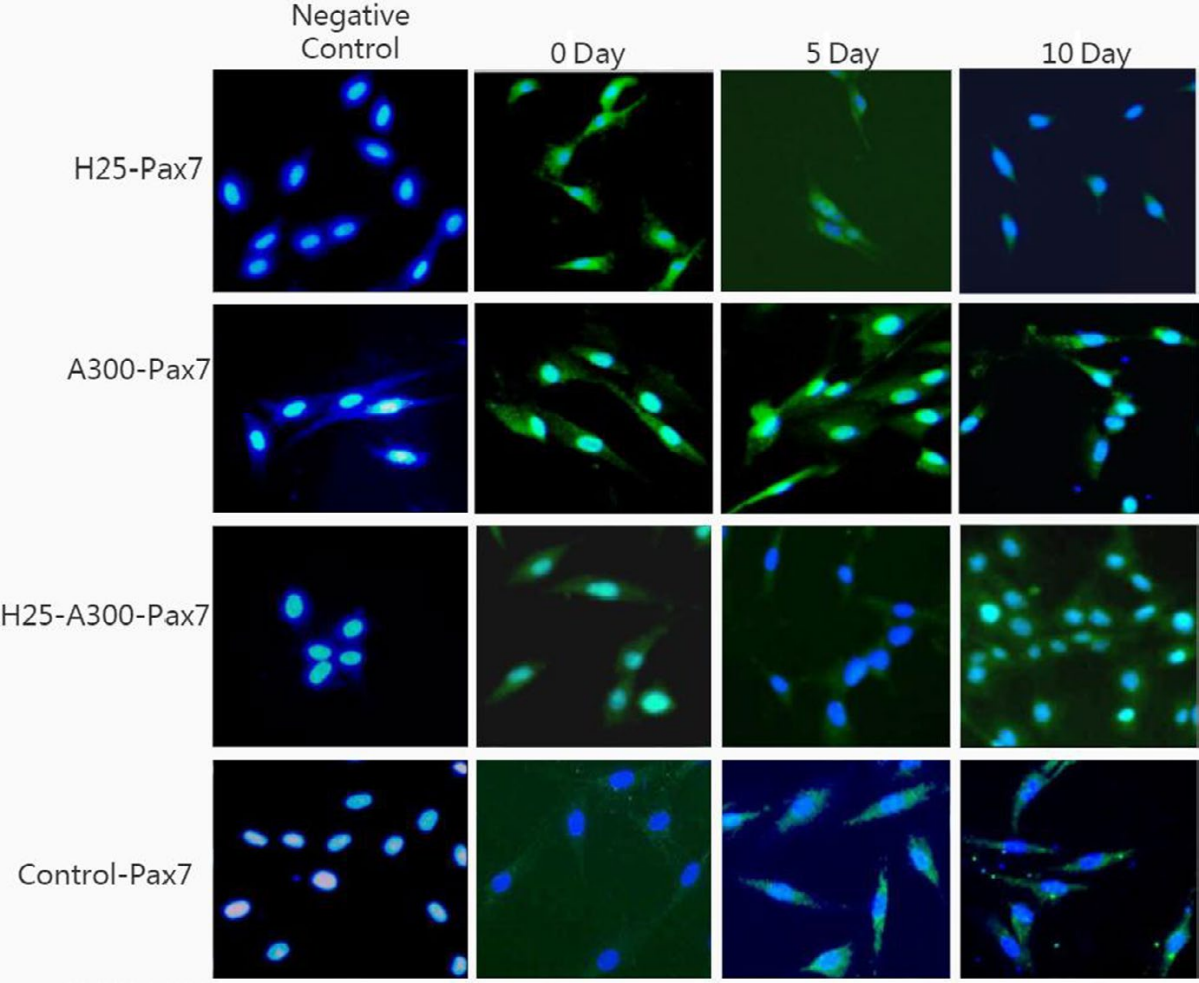
Immunofluorescence staining for Pax7 on day 0, day 5, and day 10 in AG, HG, and AG + HG, respectively. Nuclei were stained with DAPI (Magnification, ×20). AG: growth medium with 300 μM AICAR group; HG: growth medium with 25 mM glucose group; and AG + HG: growth medium with 300 μM AICAR and 25 mM glucose group

The effects of AMPK activator on the differentiation of SMSCs were assessed using a specific protein expression. As shown in Figure 1a, the phosphorylation level of the AMPK protein was significantly increased (*p* < .05) during the process, indicating that it was efficiently activated by AICAR. Pax7 expression (Figure 1b, Figure 2 of immunofluorescence), representing a specific protein of SMSCs, increased on day 7 (*p* < .05) indicating that AMPK could activate the proliferation of SMSCs. This result is in agreement with findings reported by Fu et al. (2015). The authors reported that AMPK was required for satellite cell activation and muscle regeneration by mediating the linking of the noncanonical Shh pathway to Warburg‐like glycolysis intracellular. MyoD expression (Figure 1c and Figure 3 of cellular morphology), the specific protein of SMSCs, was increased gradually to maximum in the whole process (day 10) (*p* < .05). Compared with the control group, SMSCs were converted to myoblasts when induced by 300 µM AICAR. PPARg expression, the specific protein of adipoblasts, showed no significant (*p* > .05) differences during the process (Figure 1d and Figure 4 of oil red O), while the expression of PPARg in CG was increased significantly (*p* < .05) in the middle of differentiation which was in agreement with previous studies in that satellite cells can commit spontaneously either to myogenesis or to a mesenchymal alternative differentiation program (Asakura et al., 2001; Gabi et al., 2004; Shefer, 2004). This would indicate the adipogenesis in satellite cell was inhibited by activating AMPK phosphorylation. UCP‐1 expression, the specific protein of brown adipocytes, increased during the first 7 days exhibiting a maximum level on day 7 (*p* < .05) (Figure 1e), and then decreased to the minimum on day 10 (*p* < .05), which was indicated that 300 µM AICAR induced satellite cells exhibit brown adipogenic differentiation. It had demonstrated that Myf5‐positive precursors and Myf5‐negative precursors are derived from mesenchymal stem cells (Lepper & Fan, 2010; Timmons et al., 2007) and brown adipocytes are derived from the myogenic lineage (Park, 2014), which support our present results that skeletal muscle satellite cells can commit myogenic lineage cells by activating AMPK. With the evidence of our previous study that physical exercise activated the satellite cells in longissimus dorsi (LB) of Sunit sheep by increasing Pax7 protein expression (Su et al., 2019), the present results demonstrated that the regeneration and myogenic differentiation of satellite cells can be activated by AMPK phosphorylation due to physical exercise.

### 3.2 Effects of glucose inductor on skeletal muscle satellite cell differentiation

**FIGURE 5:**
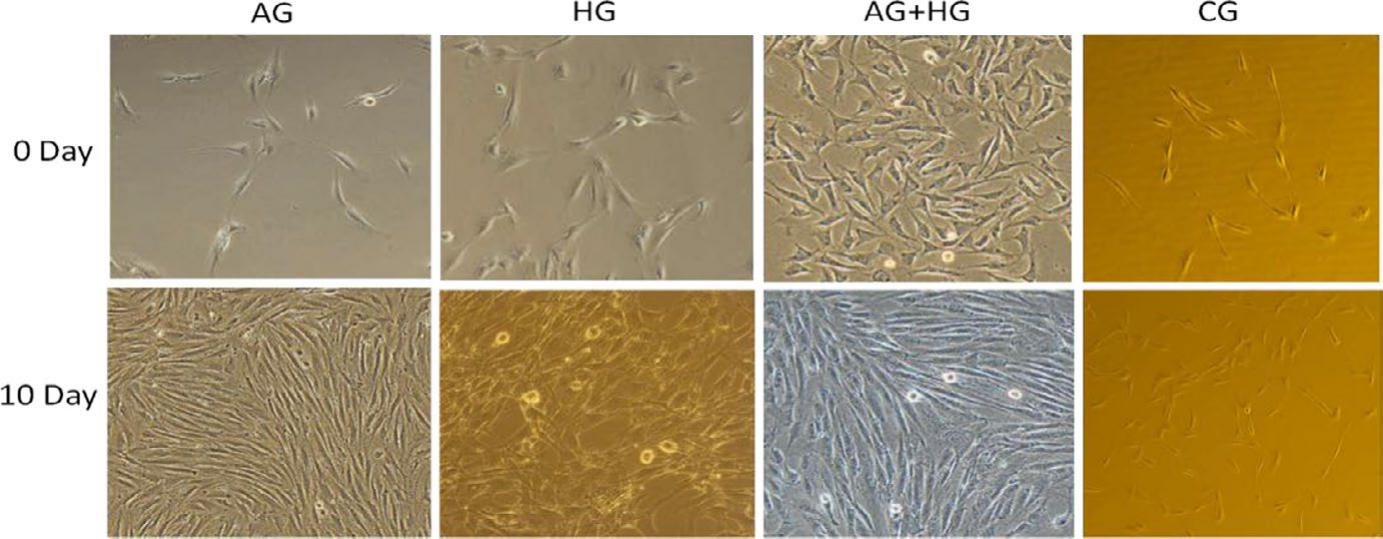
The cellular morphology of SMSCs differentiation in AG, HG, AG + HG, and CG was observed for 10 days. The images of cells were taken in day 0 and day 10 of differentiation (magnification, ×20). AG: growth medium with 300 μM AICAR group; HG: growth medium with 25 mM glucose group; AG + HG: growth medium with 300 μM AICAR and 25 mM glucose group; and CG: growth medium as control group

Dietary carbohydrates are the main energy supplements that livestock need for maintenance, growth, and production; overall glucose is the primary energy source for many animals (Nafikov & Beitz, 2007). Unlike the contribution of acetate to subcutaneous adipose, glucose was found to be preferred in intramuscular adipose synthesis, which suggests that a high concentration of intercellular glucose may increase marbling potential (Smith & Crouse, 1984). The effects of glucose inductor on differentiation of SMSCs were tested by the specific protein expression. As shown in Figure 5a and Figure 2, protein expression of Pax7 was increased in the middle of process and then deceased to the minimum (*p* < .05). This indicated that the glucose inductor inhibited proliferation of satellite cells. The protein expression of MyoD (Figure 5b and Figure 3) was similar to Pax7 that was increased in small amount and decreases to minimum in day 10 (*p* < .05), which indicating that 25 mM glucose inhibited the myogenesis of satellite cells. The protein expression of PPARg (Figure 5c) increased in day 5 (*p* < .05) and decreased afterward. Compared with the control, 25 mM glucose‐induced adipogenic differentiation of satellite cells for PPARg did not high express for whole process. As shown in Figure 4 oil red O in HG, the lipid droplet was accumulated with the process, which means that glucose indeed induces satellite cell commit adipogenesis. Thus, we test the expression of UCP‐1, the specific protein of other type of adipocytes, brown adipocytes, which showed in Figure 5d that was increased instantly in the process (*p* < .05 in day 5). Which in agree with Pasut et al. (2016), that Pax7‐null satellite cells entered brown adipogenesis other than myogenic program by downregulating MyoD and miR‐133 expression which was associated with Notch signaling rescuing. The present results suggested that the energy variation intracellular caused by high concentration of glucose may alter the fate transdifferentiation of myoblasts into brown adipocytes of skeletal muscle satellite cells.

### 3.3 Effects of glucose and AICAR cowork on skeletal muscle satellite cell differentiation

**FIGURE 6:**
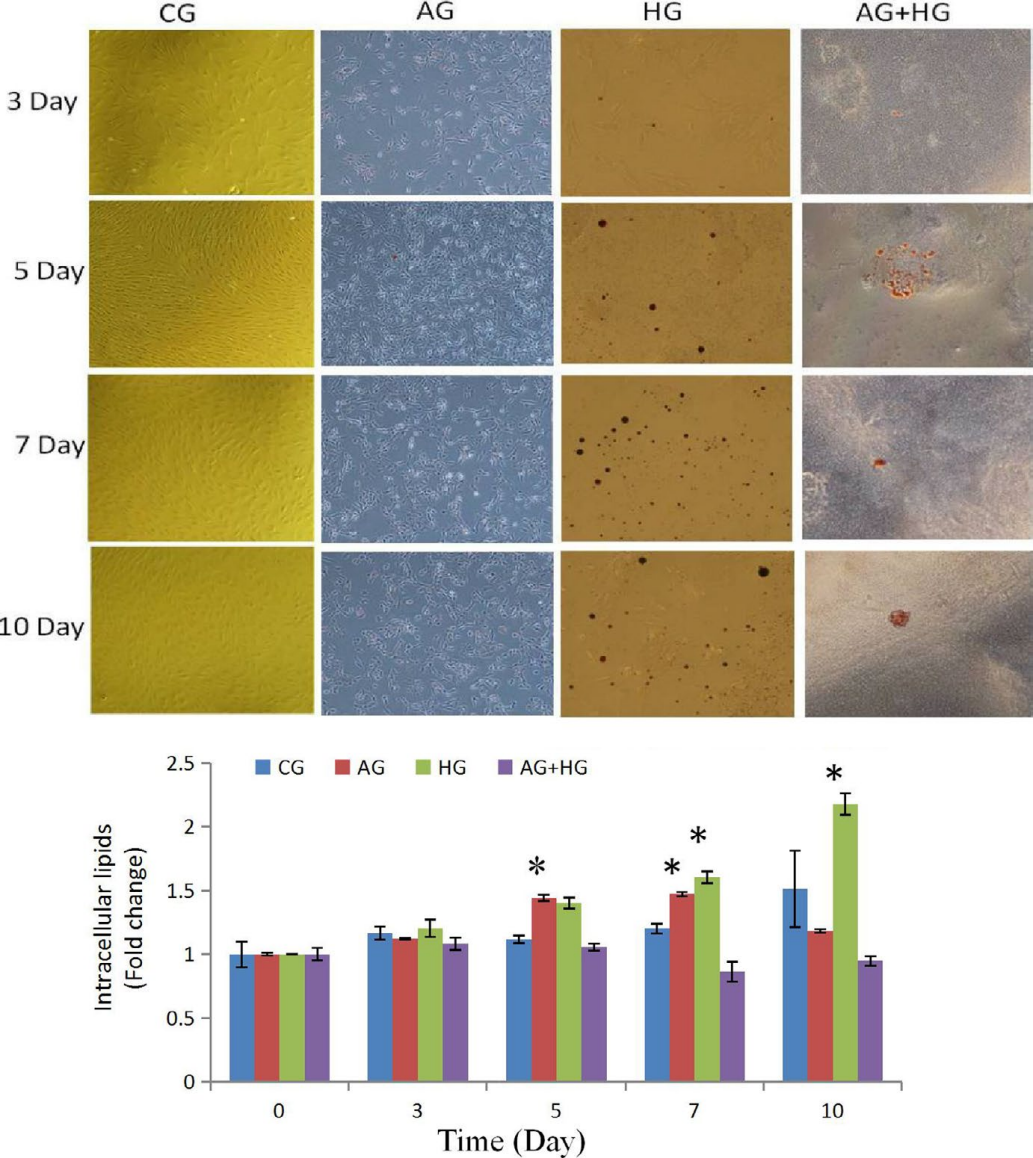
Oil Red O staining of SMSCs during the differentiation in AG, HG, AG + HG, and CG. The presence of adipocytes (lipid accumulation) was Oil Red O positive (magnification, ×10). Data are presented as mean ± SE. *Indicates a significant difference (*p* < .05) within an item compares with the undifferentiated cells (0 day) in AG and HG, respectively. AG: growth medium with 300 μM AICAR group; HG: growth medium with 25 mM glucose group; AG + HG: growth medium with 300 μM AICAR and 25 mM glucose group; and CG: growth medium as control group

To further explore the effects of the two energy sources on the differentiation of satellite cells, the SMSCs were treated concurrently with 25 mM glucose and 300 µM AMPK. Although there was no significant difference (*p* > .05), phosphorylation of AMPK was activated (Figure 6a) by glucose and induction of AICAR. Pax7 protein expression was increased in the process with no significant difference (*p* > .05) (Figure 6b), which indicated that glucose induced proliferation of satellite cells by activating AMPK. The expression of MyoD was significantly (*p* < .05) induced in days 3 and 5 (Figure 6c), but decreased in the last period. Compared with the control, the cellular morphology of SMSCs in AG + HG revealed that the two energy supplements failed to induce myogenesis of the SMSCs. In addition, the expression of PPARg showed no significant differences (*p* > .05) in the whole process (Figure 6d). Oil red O results showed (Figure 4) that two supplement inhibited lipid accumulation in the differentiation of SMSCs. Moreover, as shown in Figure 6e, UCP‐1 expression showed maximum and minimum values in days 5 and 10, respectively (*p* < .05). The previous study working on the AMPK activation and the adipogenesis in sheep fetal skeletal muscle demonstrated that AMPK activity had negative effect on the adipogenesis of fetal sheep muscle and preadipocytes (Pasut et al., 2016; Tong et al., 2008). And on the other side, Isabelle et al. (2004) found that high concentration of plasma glucose stimulated SREBP‐1c upregulated leading to an increased intracellular lipid accumulation in contracting myotubes and satellite cells. Similar treatment with glucose, Yue et al. (2016) found that satellite cells differentiate into adipogenic program by activating mTOR, while Paola et al. (2008) suggested that the oxidizing agents of reactive oxygen species (ROS) are involved in the adipogenesis of satellite cells. These evidences implied that high glucose is able to induce adipogenic differentiation of satellite cells via distinct pathway, while our results of two supplement cowork on the SMSCs suggested that glucose‐induced SMSCs committed proliferation rather than multipotential differentiation with AICAR activator. It is proposed that physical exercise with high glucose diet may not increase intracellular lipid accumulation in skeletal muscle.

## 4 CONCLUSION

In conclusion, the present study suggested that AMPK activity‐mediated satellite cell myogenic differentiation in vitro and physical exercise may activate AMPK phosphorylation to induce myogenesis of satellite cells in vivo. Furthermore, satellite cells committed adipogenesis program by increasing the concentration of intracellular glucose, which propose to enhance lipid accumulation in skeletal muscle. On the other side, variation of these two energy supplements in the meantime affected satellite cells commit to proliferation other than multipotential differentiation.

## CONFLICT OF INTEREST

The authors declare no conflicts of interest.

## ETHICAL APPROVAL

The animal experiments were approved by the Committee of Animal Experimentation and were performed under the institutional guidelines for animal experiments of the College of Animal Science, Inner Mongolian Agricultural University, China. The experiment was performed according to recommendations proposed by the European Commission (1997) to minimize the suffering of animals.

## Data Availability

Research data are not shared.
